
JOURNAL INFORMATION
==============================
NLM Title Abbreviation: Bistoury
NLM Journal ID: 0003414

The Bistoury

Publisher: Biomedical Journal Digitization Project

ARTICLE INFORMATION
==============================
PMCID: PMC9491749
PMID: 37053612
Article version: 1
Subjects: Editorial

Red Noses

Print publication date: 1872 Jul
Volume: 8
Issue: 2
First page: 56
Last page: 56
License: This work is free of known copyright restrictions. For more information, please see PMC Back Issue Digitization.
License URL: https://creativecommons.org/publicdomain/mark/1.0/

==============================

an ordinary pocket book, embraces in its vocabulary a careful selection of over 18,000 of the most important words of the language.—Prefixed to the work are tables of money, weight and measure, abbreviations, words and phrases from foreign languages, rules for spelling, explanations, etc. It is in fact a most valuable little book, and is doubly worth the dollar it costs. The publishers, Ivison, Blakeman & Co., 138 and 140 Grand Street, New York, will forward it by mail on receipt of *One Dollar*, or it can be bought at almost any book store.

RED NOSES.—A French physician, Dr. Bernier, has discovered that electricity is an efficient curative agent for the red noses of topers. This is a new and novel triumph for electricians. We fear however, that the discovery will prove an unfortunate one for the Good Templars, as its tendency may be to encourage wine bibbing, when an easy remedy is found for the resulting bloating and discoloration of the features.

A MAN'S WIFE is his best lawyer, his best counsel, his best judge, his best adviser, and also the cheapest and most reasonable.

### HENRY E. DRAKE,

DEALER IN

### DIAMONDS,

**Watches, Jewelry, Silver-Ware, French Clocks, Music Boxes and Bronzes,**

115 WATER STREET,

ELMIRA, N. Y.

All Kinds of Repairing Done Promptly.

### S. G. COMSTOCK,

DEALER IN

### HATS, CAPS, FURS,

**Gloves, Umbrellas, &c.,**

AT WHOLESALE AND RETAIL.

GENTLEMEN'S SPRING AND SUMMER HATS,

Call and see them.

The NEW STYLE HAT just received at

COMSTOCK'S,

No. 2 Rathbun Block, Elmira, N. Y.

**SMITH & HALL,**

### BANKERS,

ELMIRA, N. Y.

### MERWIN & DICKINSON,

Manufacturers and Dealers in

### HARNESS,

**Saddles, Bridles, Martingales,**

**TRUNKS, VALICES, CARPET BAGS,**

&c., &c.

**141 Water Street, Elmira, N. Y.**

### New Route!

*ELMIRA TO PHILADELPHIA. The Pennsylvania and New York Canal and Railroad Co., in connection with the Lehigh Valley Railroad Co., will run a Through Express Train, without change of cars, between Elmira and Philadelphia.*

This is the Shortest, Quickest, and only Direct Route from Elmira and Western New York, to all points in the Susquehannah, (North Branch) Wyoming and Lehigh Valleys, the Wyoming, Beaver Meadow, Hazelton and Lehigh Coal Fields, Eastern Pennsylvania, Philadelphia and New Jersey.

### LEHIGH VALLEY RAIL ROAD.

Trains leave Elmira as follows:

| | |
|---|---|
| New York Mail | 9:10 A. M. |
| Philadelphia Express | 1:00 P. M. |
| Wilkesbarre Accommodation | 2:45 P. M. |
| Towanda Accommodation | 5:30 P. M. |

Trains arrive at Elmira as follows:

| | |
|---|---|
| Towanda Acccommodation | 9:00 A. M. |
| Wilkesbarre Accommodation | 12:55 P. M. |
| Philadelphia Express | 6:15 P. M. |
| Evening Accommodation | 9:35 P. M. |

Trains leave Philadelphia via North Penn. R. R. Depot corner Berks and American Streets. Street cars run to and from all parts of the city.

From Elmira, Trains start from Erie Railway Depot. Ask for "Lehigh Valley" tickets. Through Trains Dine at White Haven, going and returning.

Connections—at Pittston with Lackawanna & Bloomsburg R. R.; at Allentown, with East Penn. R. R.; at Bethlehem, with North Penn. R. R.; at Easton, with Central Railroad of New Jersey.

Freight carried to and from Philadelphia and all intermediate points, without delay or transhipment, on Express Freight Time.

ROB'T A. SAYRE, Pres't
Penn. & N. Y. C. & R. R. Co.

Bethlehem, Pa., April, 1871.

### NORTHERN CENTRAL RAILWAY.

***DIRECT ROUTE NORTH & SOUTH.***

*To Baltimore, Washington, Philadelphia, and all points South; also to Buffalo, Niagara Falls, Suspension Bridge, Rochester, Syracuse, and all points East and West on the New York Central Railroad, and the Canadas.*

TRAINS GOING NORTH.

| | |
|---|---|
| Morning Accommodation, | 6:20 A. M. |
| Watkins Accommodation | 11:30 A. M. |
| Niagara Express | 5:00 P. M. |

TRAINS GOING SOUTH.

| | |
|---|---|
| Mail, | 5:40 A. M. |
| Niagara Express | 1:50 P. M. |
| Philadelphia Express, | 8:10 P. M. |

The Morning and Evening Accommodation northward run through to Canandaigua.

The Morning and Evening Accommodation, arriving from the North, both start from Canandaigua,

The Express Trains, both ways, are through trains between Rochester and Baltimore.

J. N. DUBARRY, General Sup't.
J. A. REDFIELD, Ass't Gen'l Sup't.
E. S. BOWEN, Division Superintendent.
